# Enrollment of Pediatric Patients in COVID-19 Interventional Trials

**DOI:** 10.1001/jamahealthforum.2023.3939

**Published:** 2023-11-22

**Authors:** Mei-Sing Ong, Ann Chen Wu, Florence T. Bourgeois

**Affiliations:** 1 Department of Population Medicine, Harvard Medical School & Harvard Pilgrim Health Care Institute, Boston, Massachusetts; 2 Department of Pediatrics, Harvard Medical School, Boston Children’s Hospital, Massachusetts; 3 Pediatric Therapeutics and Regulatory Science Initiative, Computational Health Informatics Program, Boston Children’s Hospital, Massachusetts

## Abstract

This cross-sectional study uses information gathered from ClinicalTrials.gov to assess the inclusion of children in COVID-19 interventional trials conducted in the US.

## Introduction

Children have been underrepresented in clinical research due to a number of ethical, logistical, and financial barriers. Programs have been implemented in the US to promote the inclusion of children in clinical trials.^1^ The emergence of the COVID-19 pandemic triggered a rapid investment in research activities to identify prevention measures and develop therapeutic interventions. While children were eventually determined to have a milder disease course compared with adults, studying children was critical to elucidate transmission patterns and identify treatments for pediatric patients with severe disease, including multisystem inflammatory syndrome. An assessment of the enrollment of children in COVID-19–related studies could inform policies and research programs to ensure timely and strategic inclusion of children in clinical trials during future public health emergencies. This cross-sectional study examined inclusion of children in COVID-19 interventional trials conducted in the US.

## Methods

We identified all US interventional trials studying COVID-19 and registered on ClinicalTrials.gov from January 1, 2020, to December 31, 2022. Information was extracted on trial design characteristics and trials were classified as enrolling exclusively children (age 0-17 years), both children and adults, or only adults. Detailed methods are described in eMethods in Supplement 1. This study was approved by the Harvard Pilgrim Health Care Institutional Review Board with a waiver of informed consent as data are from a publicly available source. We followed the STROBE reporting guideline. All analyses were performed in R version 4.1 (R Foundation for Statistical Computing). A 2-tailed *P* value of <.05 was considered significant.

## Results

There were 1216 COVID-19 interventional trials of which 20 (1.6%) studied exclusively children, 120 (9.9%) enrolled any children (ie, enrolled exclusively children or both children and adults), and 1096 (90.1%) enrolled only adults. The proportion of trials enrolling any children increased over time, from 45 (7.1%) in 2020 to 27 (15.7%) in 2022 (*P* = .03) (Figure).

**Figure.**
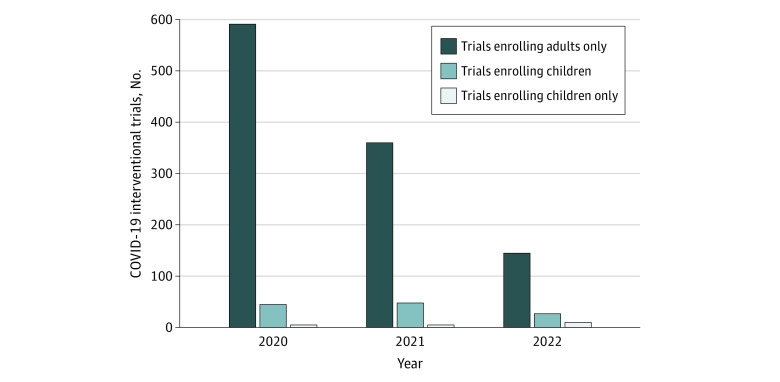
Interventional Trials Studying COVID-19, 2020 to 2022

Compared with adult-only trials, those enrolling any children were less likely to be treatment trials (48.3% vs 69.8%; *P* < .001) or to test drugs, biologics, or devices (48.3% vs 64.6%; *P* < .001). Instead, they were more likely to be prevention trials (47.5% vs 23.0%; *P* < .001) and to assess behavioral interventions (25.8% vs 16.8%; *P* = .02) and COVID-19 vaccines (14.2% vs 5.8%; *P* = .001) (Table). Fewer drug trials enrolling children were early phase trials (ie, phase 1 or 2 trials; 42.0% vs 70.4%; *P* < .001) and fewer were randomized clinical trials (69.2% vs 79.3%; *P* = .01). Among trials enrolling any children, most (71 [59.2%]) focused on children older than 2 years, with 49 (40.9%) trials open to children 2 years and younger.

**Table.** Characteristics of Interventional COVID-19 Trials

Trial attributes	No. (%)		*P* value^a^
	Trials enrolling children (n = 120)	Trials enrolling only adults (n = 1096)	
Purpose			
Prevention	57 (47.5)	252 (23.0)	<.001
Treatment	58 (48.3)	765 (69.8)	
Supportive care	5 (4.2)	79 (7.2)	
Intervention type			
Drugs and biologics	54 (45.0)	641 (58.5)	<.001
Vaccine	17 (14.2)	64 (5.8)	
Behavioral	31 (25.8)	184 (16.8)	
Device	4 (3.3)	66 (6.1)	
Diagnostic test	1 (0.8)	8 (0.7)	
Other^b^	13 (10.8)	133 (12.1)	
Trial phase^c^			
Phase 1	7 (10.1)	137 (19.5)	<.001
Phase 2^d^	22 (31.9)	357 (50.9)	
Phase 3^e^	38 (55.1)	162 (23.1)	
Phase 4	2 (2.9)	45 (6.4)	
Unspecified	0	1 (0.1)	
Early phase (phase 1 or 2) trials^c^	29 (42.0)	494 (70.4)	<.001
Assignment			
Single-arm	22 (18.3)	176 (16.1)	.61
Multiarm	98 (81.7)	920 (83.9)	
Allocation			
Randomized	83 (69.2)	869 (79.3)	.01
Masking^f^			
Open label	44 (53.0)	457 (52.6)	.93
Single-blind	10 (12.0)	95 (10.9)	
Double-blind or more	29 (34.9)	317 (36.5)	
Funding source			
Any government funding	24 (20.0)	170 (15.6)	.25
Any industry funding	57 (47.5)	479 (43.7)	.49
Enrollment size,^g^ median (IQR)	658 (165-1963)	102 (34-300)	.001
Trial status			
Ongoing	73 (60.8)	464 (42.3)	<.001
Completed	34 (28.3)	398 (36.3)	
Discontinued	13 (10.8)	199 (18.2)	
Unknown	0	35 (3.2)	
Youngest eligible age group			
Neonate (0-28 d)	32 (26.7)	NA	NA
Infant (29 d to 2 y)	17 (14.2)	NA	
Child (>2 y to 12 y)	58 (48.3)	NA	
Adolescent (>12 y to 17 y)	13 (10.8)	NA	

Abbreviation: NA, not applicable.

^a^ The reported *P* values were obtained using χ^2^ statistic for categorical variables and Kruskal-Wallis’s test for continuous variables.

^b^ Includes combination products, dietary supplements, procedures, and radiation.

^c^ Limited to trials of drugs and biologics.

^d^ Includes 56 phase 1 or 2 trials.

^e^ Includes 64 phase 2 or 3 trials.

^f^ Limited to randomized clinical trials.

^g^ Includes only completed trials.

## Discussion

Less than 10% of COVID-19 interventional trials initiated from 2020 to 2022 were open to pediatric enrollment and 1.6% enrolled exclusively children, even though children represented 18% of all reported COVID-19 cases in the US during this time period.^2^ This likely reflects established practices of delayed study of interventions in children and is in line with prior analyses indicating substantial underrepresentation of children in clinical trials even for conditions with large pediatric disease burden.^3,4^ Consistent with published data,^3^ we found that the youngest age groups were most likely to be excluded from clinical trials. A limitation of the study was the use of publicly available trial information that may be incomplete and could not be verified.

Special measures and regulatory safeguards are in place to protect children participating in research. Consequently, pediatric phase 1 studies are typically not initiated until after pivotal phase 3 trials are near completion in adults.^5^ Nonetheless, there is growing recognition of the merits and feasibility of initiating pediatric trials earlier. Recent methodologic advancements in pediatric extrapolation, pharmacokinetic and pharmacodynamic modeling, and adaptive trial designs, can be applied to support earlier initiation of pediatric trials.^6^ As part of planning efforts for future public health responses, appropriate trial designs should be identified to prioritize earlier and broader inclusion of children in clinical trials to ensure timely access to therapies.

## Notes

Supplement 1. **eMethods.** Data Extraction and Statistical Methods

Supplement 2.
Data Sharing Statement

## References

[1] Committee on Pediatric Studies Conducted Under the Best Pharmaceuticals for Children Act (BPCA) and the Pediatric Research Equity Act. In: Field MJ, Boat TF, eds. (PREA), Board on Health Sciences Policy, Institute of Medicine. Safe and Effective Medicines for Children: Pediatric Studies Conducted under the Best Pharmaceuticals for Children Act and the Pediatric Research Equity Act. National Academies Press (US); 2012, . 24872968

[2] Children and COVID-19. State data report. A joint report from the American Academy of Pediatrics and the Children’s Hospital Association. Accessed September 8, 2023. https://downloads.aap.org/AAP/PDF/AAP%20COVID-19%20State%20Data%20Report%205.11.23%20FINAL.pdf

[3] Bourgeois FT, Murthy S, Pinto C, Olson KL, Ioannidis JPA, Mandl KD. Pediatric versus adult drug trials for conditions with high pediatric disease burden. Pediatrics. 2012;130(2):285-292. doi:10.1542/peds.2012-0139 22826574 PMC3408692

[4] Wightman A, Filler G, Díaz-González de Ferris ME. The urgent need for conducting clinical trials in pediatric nephrology globally. Pediatr Nephrol. 2023;38(8):2499-2506. doi:10.1007/s00467-023-05877-2 36738331

[5] Moreno L, Pearson ADJ, Paoletti X, ; Innovative Therapies for Children with Cancer (ITCC) Consortium. Early phase clinical trials of anticancer agents in children and adolescents - an ITCC perspective. Nat Rev Clin Oncol. 2017;14(8):497-507. doi:10.1038/nrclinonc.2017.59 28508875

[6] Bhatnagar M, Sheehan S, Sharma I, . Prospect of direct benefit in pediatric trials: practical challenges and potential solutions. Pediatrics. 2021;147(5):e2020049602. doi:10.1542/peds.2020-049602 33906929 PMC8262097

