# Supplementary material for: Enrollment of Pediatric Patients in COVID-19 Interventional Trials
Source: JAMA Health Forum. 2023 Nov 22;4(11):e233939. doi: 10.1001/jamahealthforum.2023.3939 (PMC10665967; doi:10.1001/jamahealthforum.2023.3939)
Supplement: Supplement 1. — eMethods. Data Extraction and Statistical Methods [file jamahealthforum-e233939-s001.pdf]

Supplemental Online Content

Ong M, Wu AC, Bourgeios FT. Enrollment of pediatric patients in COVID-19 interventional trials. *JAMA Health Forum.* 2023;4(11):e233939. doi:10.1001/jamahealthforum.2023.3939

eMethods. Data Extraction and Statistical Methods

This supplemental material has been provided by the authors to give readers additional information about their work.

© 2023 Ong et al. *JAMA Health Forum*.

eMethods

Data extraction methods

We extracted the following trial attributes captured as structured fields in ClinicalTrials.gov: intervention type, trial phase, assignment, allocation, masking, funding source, enrollment size, trial status, and age eligibility. Two investigators reviewed the full text of the trial registrations to ascertain the purpose of the trials, classified into 3 categories: treatment, prevention, and supportive care. Disagreements between investigators were resolved by consensus. For trials enrolling children, we further identified the youngest eligible age group, classified into the following categories: neonate (0 – 28 days), infant (29 days – 2 years), child (>2 years – 12 years), and adolescent (>12 years – 17 years). We classified trial funding sources as “any government funding” and “any industry funding” based on mention of these funding types in the list of funders included for each trial. Finally, intervention type was classified as drugs and biologics (excluding vaccine trials), vaccines, behavioral, device, diagnostic test, and other. This study was approved by the Harvard Pilgrim Health Care Institutional Review Board and follows the STROBE reporting guideline for cross-sectional studies.

Statistical methods

We compared characteristics of trials enrolling any children and those enrolling only adults using Chi-square and Kruskal-Wallis tests. Temporal trends were assessed using linear regression analysis. All analyses were performed in R Statistical Software (version 4.1). A two-tailed p-value of <0.05 was considered significant.

© 2023 Ong et al. *JAMA Health Forum*.
