# Supplementary material for: Enrollment of Pediatric Patients in COVID-19 Interventional Trials
Source: JAMA Health Forum. 2023 Nov 22;4(11):e233939. doi: 10.1001/jamahealthforum.2023.3939 (PMC10665967; doi:10.1001/jamahealthforum.2023.3939)
Supplement: Supplement 2. — Data Sharing Statement [file jamahealthforum-e233939-s002.pdf]

Data Sharing Statement

Ong. Enrollment of Pediatric Patients in COVID-19 Interventional Trials. *JAMA Health Forum*. Published November 22, 2023. doi:10.1001/jamahealthforum.2023.3939

Data
Data available: Yes
Data types: Data (not involving human participants)
How to access data: All data used in our study are available via ct.gov. Additional variables used in our study can be provided upon request (Mei-Sing_Ong@hms.harvard.edu).
When available: With publication

Supporting Documents
Document types: None

Additional Information
Who can access the data: researchers whose proposed use of the data has been approved
Types of analyses: for any purpose
Mechanisms of data availability: after approval of a proposal
